# Supplementary material for: Novel Cerium(IV) Coordination Compounds of Monensin and Salinomycin
Source: Molecules. 2023 Jun 9;28(12):4676. doi: 10.3390/molecules28124676 (PMC10301318; doi:10.3390/molecules28124676)
Supplement: Supplementary file 1 [file molecules-28-04676-s001.zip › molecules-2432340-supplementary.pdf]

Article

Novel Cerium(IV) Coordination Compounds of Monensin and Salinomycin

Nikolay Petkov [1,*], Ivayla Pantcheva [1,*], Anela Ivanova [1], Radostina Stoyanova [2], Rositsa Kukeva [2], Radostina Alexandrova [3], Abedullkader Abudalleh [3] and Petar Dorkov [4]

[1] Faculty of Chemistry and Pharmacy, Sofia University St. Kliment Ohridski, 1164 Sofia, Bulgaria; aivanova@chem.uni-sofia.bg

[2] Institute of General and Inorganic Chemistry, Bulgarian Academy of Sciences, 1113 Sofia, Bulgaria; radstoy@svr.igic.bas.bg (R.S.); rositsakukeva@yahoo.com (R.K.)

[3] Institute of Experimental Morphology, Pathology and Anthropology with Museum, Bulgarian Academy of Sciences, 1113 Sofia, Bulgaria; rialexandrova@hotmail.com (R.A.); alkader78mah@yahoo.com (A.A.)

[4] Research and Development Department, Biovet Ltd., 4550 Peshtera, Bulgaria; p_dorkov@abv.bg

* Correspondence: ahnp@chem.uni-sofia.bg (N.P.); ipancheva@chem.uni-sofia.bg (I.P.); Tel.: +359-2-8161446 (N.P. & I.P.)

Supplementary Information

Citation: Petkov, N.; Pantcheva, I.; Ivanova, A.; Stoyanova, R.; Kukeva, R.; Alexandrova, R.; Abudalleh, A.; Dorkov, P. Novel Cerium(IV) Coordination Compounds of Monensin and Salinomycin. *Molecules* **2023**, *28*, 4676. doi.org/10.3390/molecules28124676

Academic Editor: Mihaela Badea

Received: 18 May 2023
Revised: 1 June 2023
Accepted: 7 June 2023
Published: 9 June 2023

Copyright: © 2023 by the authors. Licensee MDPI, Basel, Switzerland. This article is an open access article distributed under the terms and conditions of the Creative Commons Attribution (CC BY) license (https://creativecommons.org/licenses/by/4.0/).

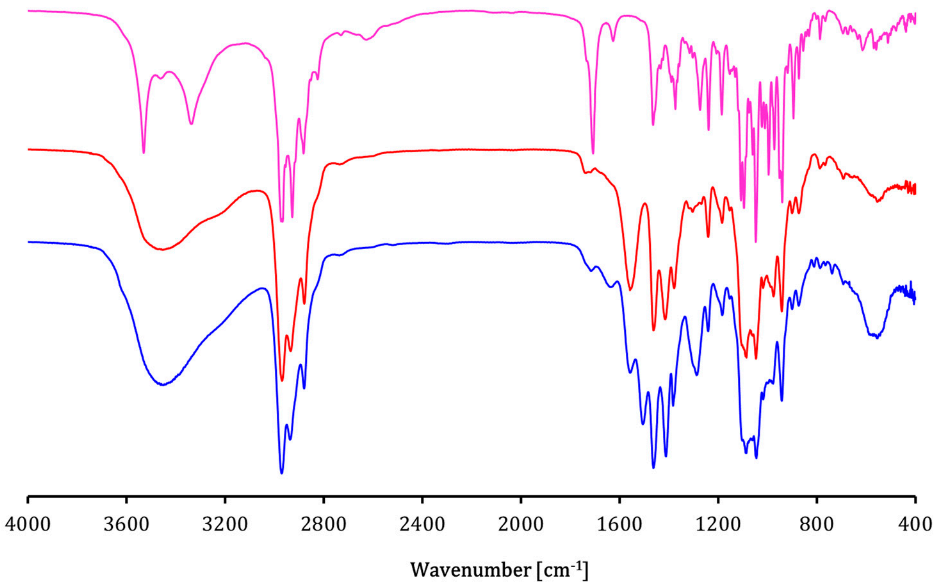

Figure S1. IR spectra of monensic acid (magenta), complex **1a** (red) and complex **2a** (blue).

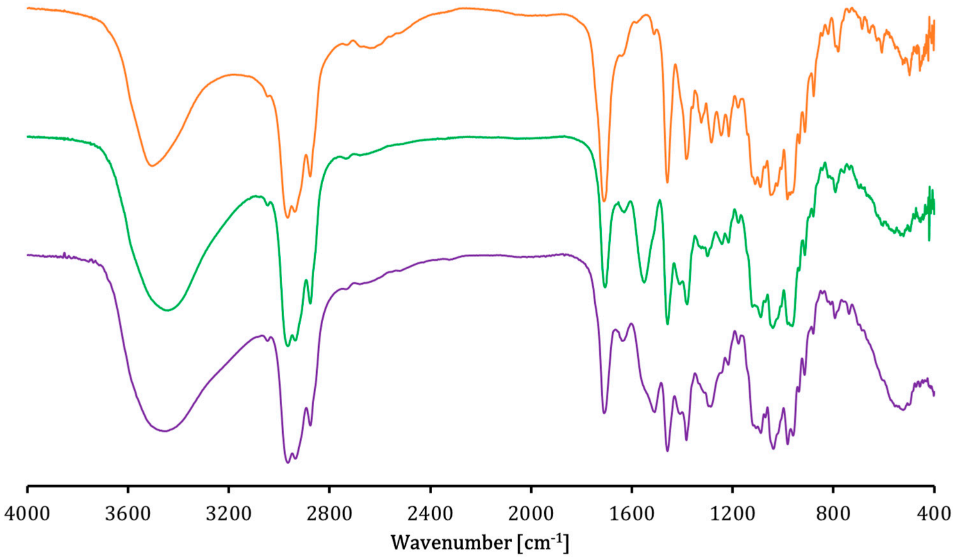

Figure S2. IR spectra of salinomycinic acid (orange), complex **1b** (green) and complex **2b** (violet).

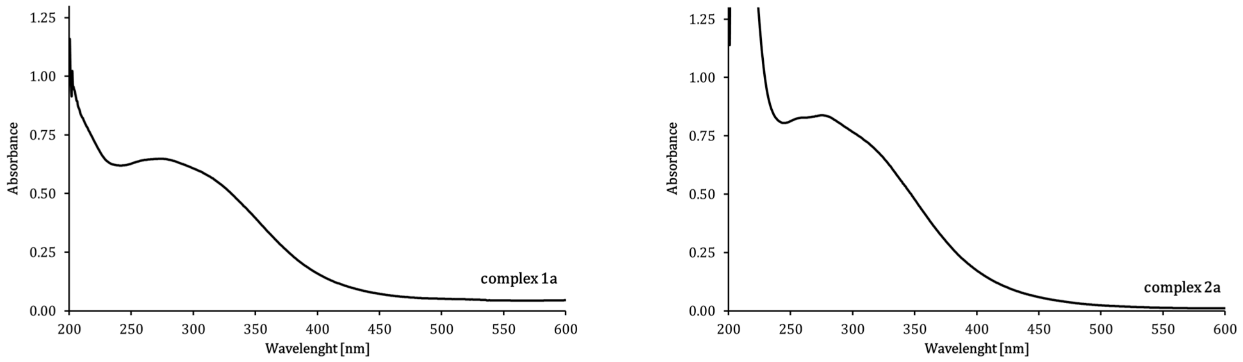

Figure S3. UV-Vis spectra of **1a** and **2a** in MeOH at concentration 0.3125 mg/mL.

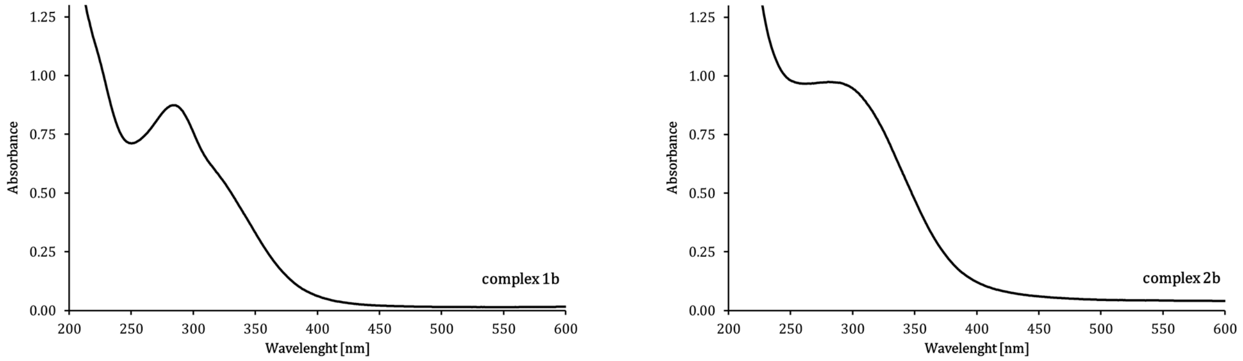

Figure S4. UV-Vis spectra of **1b** and **2b** in MeOH at concentration 0.3125 mg/mL.

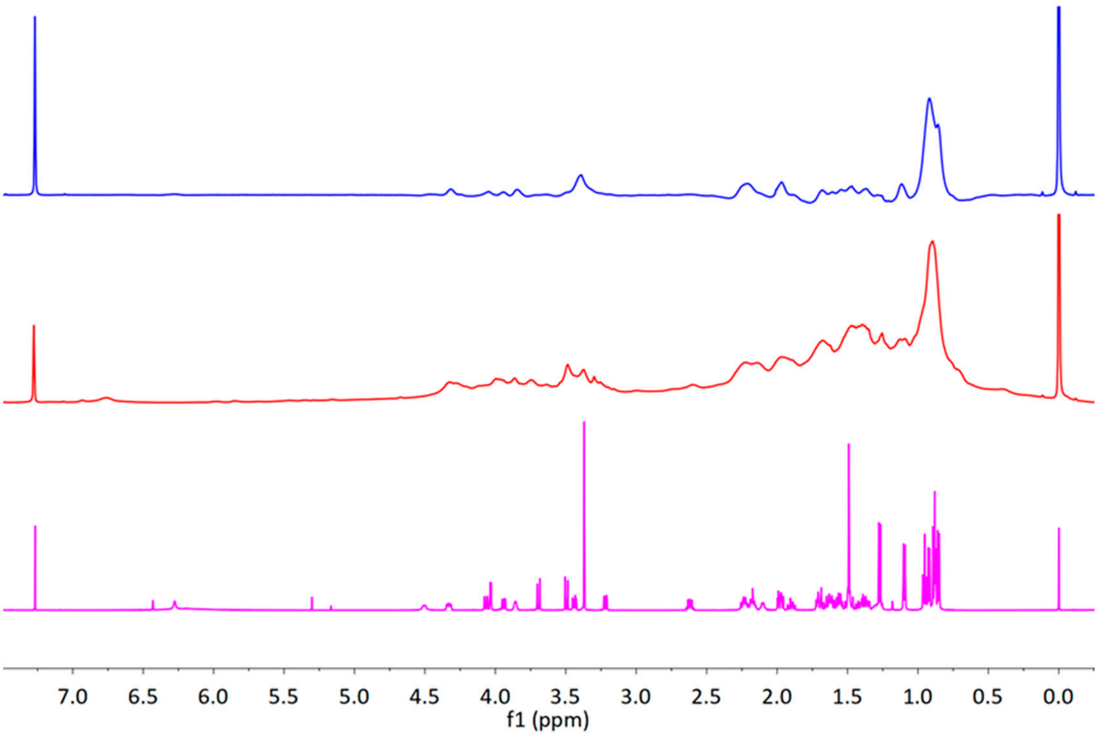

Figure S5. ^{1}H-NMR spectra of monensic acid (magenta) and complexes **1a** (red) / **2a** (blue).

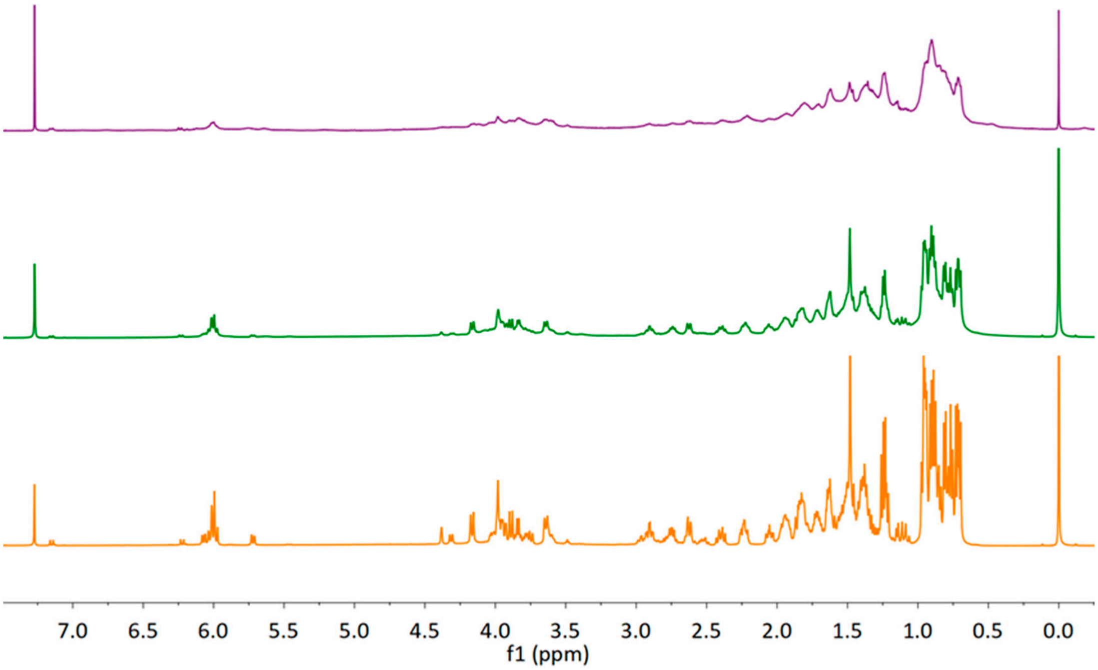

Figure S6. ^{1}H-NMR spectra of salinomycinic acid (orange) and complexes **1b** (green) / **2b** (purple).

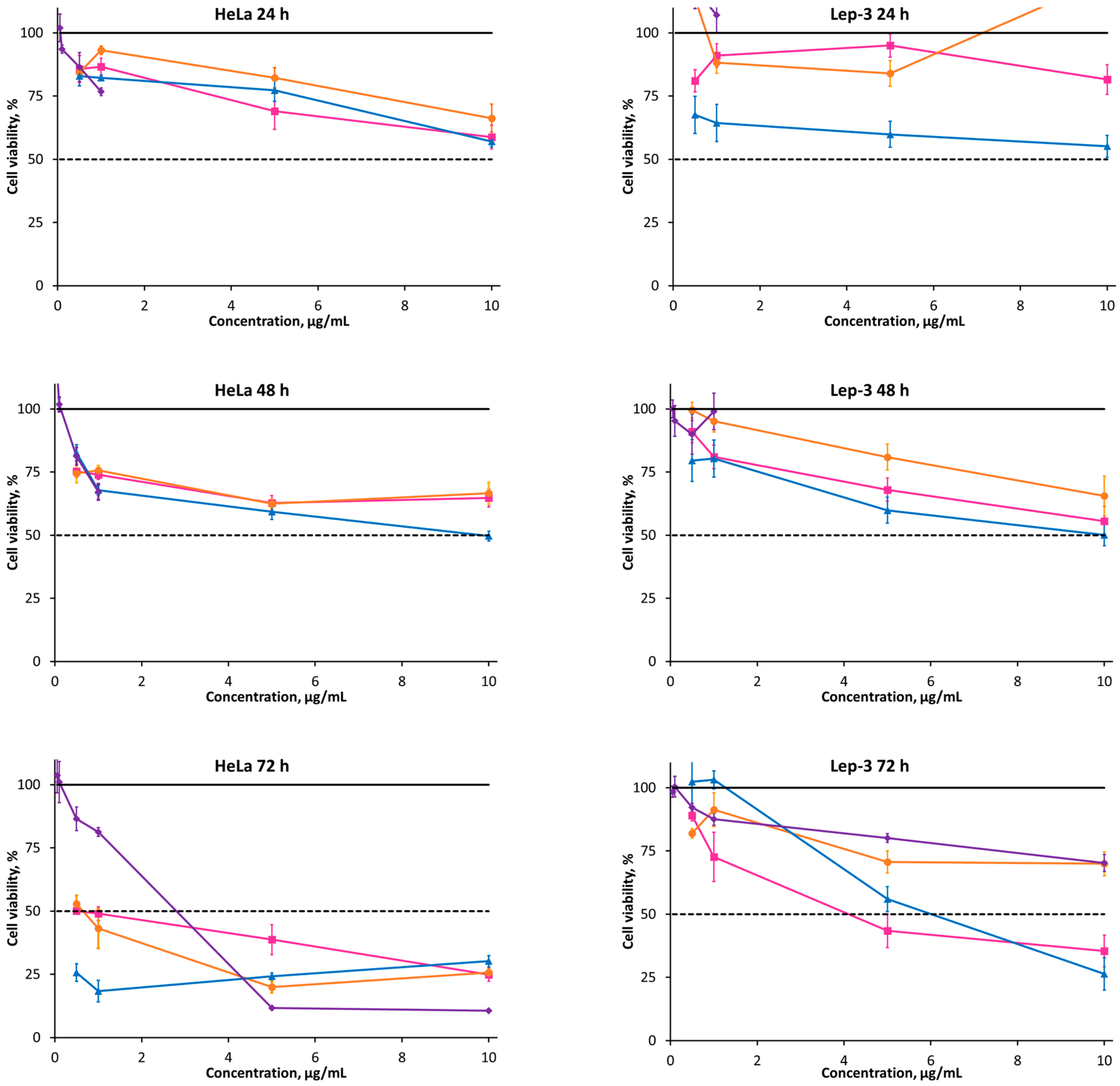

Figure S7. Cell viability of (**a**) HeLa and (**b**) Lep-3 after 24-72 h treatment with Mon×H_2O, SalH and complexes **2a**-**b** (MTT test). Colour code: Mon×H_2O – magenta, SalH – orange, **2a** – blue, **2b** – purple.

Table S1. Coordinates of Monensinate anion.

Residue name	Atom number	System numbering	x	y	z
1MNA	O1	1	-0.223	0.21	0.133
1MNA	C1	2	-0.306	0.12	0.192
1MNA	O2	3	-0.259	0.037	0.269
1MNA	C2	4	-0.457	0.133	0.171
1MNA	H1	5	-0.504	0.06	0.237
1MNA	C36	6	-0.504	0.273	0.215
1MNA	H59	7	-0.469	0.292	0.317
1MNA	H60	8	-0.613	0.278	0.215
1MNA	H61	9	-0.464	0.349	0.148
1MNA	C3	10	-0.501	0.102	0.025
1MNA	H2	11	-0.461	0.182	-0.038
1MNA	O11	12	-0.644	0.111	0.025
1MNA	C35	13	-0.7	0.155	-0.098
1MNA	H56	14	-0.661	0.254	-0.124
1MNA	H57	15	-0.808	0.163	-0.086
1MNA	H58	16	-0.679	0.085	-0.178
1MNA	C4	17	-0.459	-0.036	-0.033
1MNA	H3	18	-0.521	-0.054	-0.121
1MNA	C34	19	-0.491	-0.151	0.065
1MNA	H53	20	-0.48	-0.247	0.015
1MNA	H54	21	-0.594	-0.142	0.1
1MNA	H55	22	-0.423	-0.148	0.15
1MNA	C5	23	-0.312	-0.045	-0.083
1MNA	H4	24	-0.247	-0.038	0.004
1MNA	C6	25	-0.269	0.064	-0.185
1MNA	H5	26	-0.267	0.16	-0.134
1MNA	C33	27	-0.364	0.076	-0.306
1MNA	H48	28	-0.379	-0.021	-0.352
1MNA	H49	29	-0.324	0.145	-0.379
1MNA	H50	30	-0.461	0.115	-0.273
1MNA	C7	31	-0.125	0.038	-0.235
1MNA	H6	32	-0.102	0.107	-0.316
1MNA	O10	33	-0.033	0.06	-0.131
1MNA	H52	34	-0.031	-0.021	-0.078
1MNA	C8	35	-0.108	-0.107	-0.285
1MNA	H7	36	-0.003	-0.126	-0.305
1MNA	H8	37	-0.164	-0.119	-0.378
1MNA	O3	38	-0.294	-0.175	-0.141
1MNA	C9	39	-0.161	-0.208	-0.183
1MNA	C10	40	-0.155	-0.353	-0.229
1MNA	H9	41	-0.067	-0.37	-0.29
1MNA	H10	42	-0.245	-0.38	-0.284
1MNA	C11	43	-0.145	-0.428	-0.096
1MNA	H11	44	-0.092	-0.523	-0.107
1MNA	H12	45	-0.246	-0.446	-0.058

1MNA	O4	46	-0.087	-0.202	-0.061
1MNA	C12	47	-0.071	-0.33	-0.004
1MNA	C32	48	-0.134	-0.327	0.136
1MNA	H45	49	-0.086	-0.251	0.196
1MNA	H46	50	-0.122	-0.424	0.184
1MNA	H47	51	-0.24	-0.303	0.128
1MNA	C13	52	0.081	-0.361	0.008
1MNA	H13	53	0.092	-0.458	0.057
1MNA	C14	54	0.161	-0.363	-0.122
1MNA	H14	55	0.144	-0.454	-0.179
1MNA	H15	56	0.139	-0.274	-0.18
1MNA	C15	57	0.303	-0.356	-0.068
1MNA	H16	58	0.367	-0.307	-0.141
1MNA	H17	59	0.341	-0.456	-0.048
1MNA	O5	60	0.149	-0.264	0.085
1MNA	C16	61	0.29	-0.275	0.062
1MNA	C30	62	0.348	-0.353	0.183
1MNA	H40	63	0.303	-0.453	0.184
1MNA	H41	64	0.455	-0.367	0.169
1MNA	C31	65	0.323	-0.289	0.32
1MNA	H42	66	0.373	-0.192	0.326
1MNA	H43	67	0.363	-0.353	0.398
1MNA	H44	68	0.216	-0.276	0.336
1MNA	C17	69	0.345	-0.13	0.052
1MNA	H18	70	0.301	-0.074	0.135
1MNA	C18	71	0.498	-0.107	0.061
1MNA	H19	72	0.533	-0.12	0.163
1MNA	C29	73	0.587	-0.189	-0.034
1MNA	H37	74	0.557	-0.174	-0.138
1MNA	H38	75	0.581	-0.295	-0.011
1MNA	H39	76	0.691	-0.158	-0.023
1MNA	C19	77	0.501	0.04	0.022
1MNA	H20	78	0.477	0.101	0.109
1MNA	H21	79	0.598	0.071	-0.019
1MNA	O6	80	0.307	-0.062	-0.067
1MNA	C20	81	0.39	0.051	-0.084
1MNA	H22	82	0.435	0.041	-0.183
1MNA	C21	83	0.31	0.183	-0.08
1MNA	H23	84	0.233	0.177	-0.157
1MNA	O7	85	0.248	0.194	0.048
1MNA	C25	86	0.165	0.308	0.069
1MNA	O8	87	0.056	0.308	-0.021
1MNA	H30	88	0.033	0.216	-0.04
1MNA	C26	89	0.109	0.294	0.212
1MNA	H28	90	0.191	0.304	0.283
1MNA	H29	91	0.036	0.373	0.23
1MNA	O9	92	0.047	0.168	0.23
1MNA	H51	93	0.106	0.101	0.192

1MNA	C24	94	0.25	0.436	0.052
1MNA	H27	95	0.329	0.434	0.127
1MNA	C27	96	0.17	0.567	0.071
1MNA	H31	97	0.236	0.652	0.056
1MNA	H32	98	0.129	0.573	0.171
1MNA	H33	99	0.089	0.572	-0.001
1MNA	C23	100	0.316	0.436	-0.087
1MNA	H25	101	0.382	0.523	-0.095
1MNA	H26	102	0.239	0.443	-0.164
1MNA	C22	103	0.398	0.308	-0.107
1MNA	H24	104	0.48	0.309	-0.035
1MNA	C28	105	0.458	0.309	-0.249
1MNA	H34	106	0.378	0.301	-0.323
1MNA	H35	107	0.528	0.226	-0.261
1MNA	H36	108	0.513	0.402	-0.265

Table S2. Force field parameters of Monensinate anion.

Atom number	Atom type	Charge	System numbering
O1	opls_272	-0.75932	1
C1	opls_271	0.767858	2
O2	opls_272	-0.75932	3
C2	opls_275	0.011776	4
H1	opls_140	-0.01005	5
C36	opls_135	-0.03248	6
H59	opls_140	-0.01009	7
H60	opls_140	-0.01009	8
H61	opls_140	-0.01009	9
C3	opls_183	-5.2E-05	10
H2	opls_185	0.062316	11
O11	opls_180	-0.37608	12
C35	opls_181	0.01222	13
H56	opls_185	0.054062	14
H57	opls_185	0.054062	15
H58	opls_185	0.054062	16
C4	opls_137	-0.01237	17
H3	opls_140	0.023877	18
C34	opls_135	-0.01507	19
H53	opls_140	-0.01507	20
H54	opls_140	-0.01507	21
H55	opls_140	-0.01507	22
C5	opls_183	-0.03804	23
H4	opls_185	0.113645	24
C6	opls_137	0.005747	25
H5	opls_140	0.041605	26
C33	opls_135	-0.03634	27
H48	opls_140	0.017833	28

H49	opls_140	0.017833	29
H50	opls_140	0.017833	30
C7	opls_158	-0.00614	31
H6	opls_156	0.118947	32
O10	opls_154	-0.52261	33
H52	opls_155	0.269562	34
C8	opls_136	-0.05577	35
H7	opls_140	0.062372	36
H8	opls_140	0.062372	37
O3	opls_186	-0.1705	38
C9	opls_197	0.011581	39
C10	opls_136	-0.06121	40
H9	opls_140	0.056471	41
H10	opls_140	0.056471	42
C11	opls_136	-0.0362	43
H11	opls_140	0.052219	44
H12	opls_140	0.052219	45
O4	opls_186	-0.08729	46
C12	opls_184	0.02996	47
C32	opls_135	-0.06965	48
H45	opls_140	0.032349	49
H46	opls_140	0.032349	50
H47	opls_140	0.032349	51
C13	opls_183	-0.00168	52
H13	opls_185	0.072529	53
C14	opls_136	-0.01932	54
H14	opls_140	0.040879	55
H15	opls_140	0.040879	56
C15	opls_136	-0.03757	57
H16	opls_140	0.045105	58
H17	opls_140	0.045105	59
O5	opls_180	-0.24735	60
C16	opls_183	-0.00586	61
C30	opls_136	0.011472	62
H40	opls_140	0.029762	63
H41	opls_140	0.029762	64
C31	opls_135	-0.03578	65
H42	opls_140	0.012174	66
H43	opls_140	0.012174	67
H44	opls_140	0.012174	68
C17	opls_183	-0.00825	69
H18	opls_185	0.130474	70
C18	opls_137	0.002691	71
H19	opls_140	0.048977	72
C29	opls_135	-0.04092	73
H37	opls_140	0.006976	74
H38	opls_140	0.006976	75
H39	opls_140	0.006976	76

C19	opls_136	-0.00354	77
H20	opls_140	0.040868	78
H21	opls_140	0.040868	79
O6	opls_180	-0.32153	80
C20	opls_183	0.011997	81
H22	opls_185	0.072994	82
C21	opls_183	-0.03497	83
H23	opls_185	0.078201	84
O7	opls_180	-0.14321	85
C25	opls_198	0.085386	86
O8	opls_187	-0.5059	87
H30	opls_188	0.394475	88
C26	opls_157	0.023082	89
H28	opls_156	0.091	90
H29	opls_156	0.091	91
O9	opls_154	-0.63279	92
H51	opls_155	0.436206	93
C24	opls_137	0.011717	94
H27	opls_140	0.024966	95
C27	opls_135	-0.07169	96
H31	opls_140	0.023726	97
H32	opls_140	0.023726	98
H33	opls_140	0.023726	99
C23	opls_136	-0.02672	100
H25	opls_140	0.040197	101
H26	opls_140	0.040197	102
C22	opls_137	0.020264	103
H24	opls_140	0.036147	104
C28	opls_135	-0.1118	105
H34	opls_140	0.027687	106
H35	opls_140	0.027687	107
H36	opls_140	0.027687	108

Table S3. Coordinates of Salinomycinate anion.

Residue name	Atom number	System numbering	x	y	z
1SLA	O1	1	1.248	0.772	1.166
1SLA	C1	2	1.183	0.678	1.207
1SLA	O2	3	1.208	0.553	1.17
1SLA	C2	4	1.069	0.69	1.304
1SLA	H1	5	1.064	0.783	1.336
1SLA	C41	6	1.091	0.598	1.423
1SLA	H65	7	1.016	0.606	1.484
1SLA	H66	8	1.093	0.506	1.392
1SLA	C42	9	1.217	0.625	1.499
1SLA	H67	10	1.293	0.614	1.441
1SLA	H68	11	1.225	0.563	1.572

1SLA	H69	12	1.215	0.715	1.533
1SLA	C3	13	0.938	0.656	1.232
1SLA	H2	14	0.929	0.559	1.232
1SLA	O3	15	0.938	0.698	1.096
1SLA	C4	16	0.815	0.711	1.306
1SLA	H3	17	0.736	0.664	1.276
1SLA	H4	18	0.825	0.695	1.401
1SLA	C5	19	0.796	0.859	1.282
1SLA	H5	20	0.869	0.908	1.324
1SLA	H6	21	0.713	0.888	1.323
1SLA	C6	22	0.793	0.888	1.132
1SLA	H7	23	0.788	0.985	1.12
1SLA	C40	24	0.669	0.825	1.065
1SLA	H62	25	0.65	0.872	0.984
1SLA	H63	26	0.594	0.831	1.125
1SLA	H64	27	0.687	0.733	1.046
1SLA	C7	28	0.924	0.84	1.071
1SLA	H8	29	0.998	0.887	1.114
1SLA	C8	30	0.935	0.854	0.921
1SLA	H9	31	0.86	0.804	0.881
1SLA	C39	32	0.921	1	0.877
1SLA	H59	33	0.988	1.053	0.922
1SLA	H60	34	0.834	1.033	0.9
1SLA	H61	35	0.934	1.007	0.783
1SLA	C9	36	1.064	0.792	0.869
1SLA	H10	37	1.074	0.702	0.908
1SLA	O4	38	1.179	0.87	0.907
1SLA	H11	39	1.207	0.843	0.98
1SLA	C10	40	1.074	0.779	0.717
1SLA	H12	41	1.09	0.868	0.679
1SLA	C38	42	0.948	0.719	0.654
1SLA	H56	43	0.964	0.702	0.561
1SLA	H57	44	0.875	0.782	0.663
1SLA	H58	45	0.925	0.637	0.699
1SLA	C11	46	1.186	0.687	0.677
1SLA	O5	47	1.22	0.592	0.745
1SLA	C12	48	1.252	0.711	0.541
1SLA	H13	49	1.182	0.745	0.482
1SLA	C36	50	1.301	0.58	0.479
1SLA	H51	51	1.365	0.6	0.409
1SLA	H52	52	1.345	0.527	0.547
1SLA	C37	53	1.187	0.498	0.42
1SLA	H53	54	1.13	0.467	0.49
1SLA	H54	55	1.223	0.423	0.372
1SLA	H55	56	1.136	0.553	0.36
1SLA	C13	57	1.36	0.821	0.551
1SLA	H14	58	1.421	0.811	0.475
1SLA	O6	59	1.285	0.943	0.535

1SLA	C14	60	1.442	0.823	0.678
1SLA	H15	61	1.381	0.825	0.755
1SLA	C35	62	1.533	0.7	0.689
1SLA	H48	63	1.479	0.621	0.699
1SLA	H49	64	1.59	0.709	0.766
1SLA	H50	65	1.587	0.693	0.61
1SLA	C15	66	1.526	0.95	0.68
1SLA	H16	67	1.575	0.955	0.763
1SLA	H17	68	1.59	0.947	0.607
1SLA	C16	69	1.439	1.074	0.665
1SLA	H18	70	1.376	1.075	0.74
1SLA	C34	71	1.522	1.203	0.672
1SLA	H45	72	1.564	1.21	0.758
1SLA	H46	73	1.464	1.279	0.659
1SLA	H47	74	1.589	1.202	0.604
1SLA	C17	75	1.359	1.066	0.535
1SLA	O7	76	1.26	1.166	0.544
1SLA	C18	77	1.442	1.082	0.412
1SLA	H19	78	1.507	1.018	0.394
1SLA	C19	79	1.428	1.182	0.328
1SLA	H20	80	1.486	1.189	0.256
1SLA	C20	81	1.324	1.284	0.346
1SLA	H21	82	1.361	1.358	0.398
1SLA	O8	83	1.278	1.336	0.222
1SLA	H22	84	1.227	1.282	0.187
1SLA	C21	85	1.21	1.223	0.425
1SLA	O9	86	1.15	1.13	0.34
1SLA	C22	87	1.099	1.322	0.466
1SLA	H23	88	1.103	1.34	0.562
1SLA	H24	89	1.108	1.406	0.418
1SLA	C23	90	0.973	1.254	0.43
1SLA	H25	91	0.916	1.315	0.381
1SLA	H26	92	0.926	1.228	0.511
1SLA	C24	93	1.004	1.132	0.346
1SLA	C33	94	0.96	1.002	0.411
1SLA	H42	95	0.864	1.002	0.419
1SLA	H43	96	0.999	0.995	0.498
1SLA	H44	97	0.988	0.928	0.357
1SLA	C25	98	0.958	1.141	0.203
1SLA	H27	99	0.996	1.066	0.153
1SLA	C26	100	0.807	1.139	0.186
1SLA	H28	101	0.767	1.208	0.242
1SLA	H29	102	0.772	1.053	0.215
1SLA	C27	103	0.768	1.163	0.041
1SLA	H30	104	0.798	1.088	-0.013
1SLA	H31	105	0.672	1.169	0.034
1SLA	C28	106	0.831	1.29	-0.014
1SLA	O11	107	0.784	1.403	0.059

1SLA	H36	108	0.702	1.405	0.058
1SLA	C31	109	0.797	1.305	-0.161
1SLA	H37	110	0.7	1.304	-0.171
1SLA	H38	111	0.832	1.229	-0.209
1SLA	C32	112	0.851	1.434	-0.228
1SLA	H39	113	0.817	1.51	-0.181
1SLA	H40	114	0.822	1.437	-0.319
1SLA	H41	115	0.947	1.434	-0.224
1SLA	C29	116	0.983	1.288	0.01
1SLA	H32	117	1.016	1.378	-0.01
1SLA	C30	118	1.058	1.191	-0.078
1SLA	H33	119	1.148	1.181	-0.045
1SLA	H34	120	1.061	1.225	-0.168
1SLA	H35	121	1.014	1.106	-0.078
1SLA	O10	122	1.012	1.262	0.15

Table S4. Force field parameters of Monensinate anion.

Atom number	Atom type	Charge	System numbering
O1	opls_272	-0.77241	1
C1	opls_271	0.752127	2
O2	opls_272	-0.77241	3
C2	opls_275	0.010986	4
H1	opls_140	-0.01064	5
C41	opls_136	0.021543	6
H65	opls_140	-0.00345	7
H66	opls_140	-0.00345	8
C42	opls_135	-0.03829	9
H67	opls_140	-0.01281	10
H68	opls_140	-0.01281	11
H69	opls_140	-0.01281	12
C3	opls_183	0.037095	13
H2	opls_185	0.030142	14
O3	opls_180	-0.20186	15
C4	opls_136	-0.01487	16
H3	opls_140	0.030084	17
H4	opls_140	0.030084	18
C5	opls_136	-0.01398	19
H5	opls_140	-0.00716	20
H6	opls_140	-0.00716	21
C6	opls_137	0.029097	22
H7	opls_140	0.06938	23
C40	opls_135	-0.13074	24
H62	opls_140	0.021503	25
H63	opls_140	0.021503	26
H64	opls_140	0.021503	27
C7	opls_183	-0.12583	28

H8	opls_185	0.065625	29
C8	opls_137	0.023869	30
H9	opls_140	-0.01505	31
C39	opls_135	-0.10723	32
H59	opls_140	0.029155	33
H60	opls_140	0.029155	34
H61	opls_140	0.029155	35
C9	opls_158	0.006664	36
H10	opls_140	0.129822	37
O4	opls_154	-0.49483	38
H11	opls_155	0.358849	39
C10	opls_137	0.055634	40
H12	opls_140	0.113075	41
C38	opls_135	-1.16238	42
H56	opls_140	0.334	43
H57	opls_140	0.334	44
H58	opls_140	0.334	45
C11	opls_280	0.3848	46
O5	opls_281	-0.43285	47
C12	opls_137	-0.03204	48
H13	opls_140	0.040012	49
C36	opls_136	0.014232	50
H51	opls_140	0.016055	51
H52	opls_140	0.016055	52
C37	opls_135	-0.0436	53
H53	opls_140	0.013147	54
H54	opls_140	0.013147	55
H55	opls_140	0.013147	56
C13	opls_183	-0.03602	57
H14	opls_185	0.165285	58
O6	opls_186	-0.19965	59
C14	opls_137	-0.00522	60
H15	opls_140	0.02111	61
C35	opls_135	-0.03584	62
H48	opls_140	0.012371	63
H49	opls_140	0.012371	64
H50	opls_140	0.012371	65
C15	opls_137	-0.01871	66
H16	opls_140	0.027201	67
H17	opls_140	0.027201	68
C16	opls_137	-0.01777	69
H18	opls_140	0.084283	70
C34	opls_135	-0.03005	71
H45	opls_140	0.008884	72
H46	opls_140	0.008884	73
H47	opls_140	0.008884	74
C17	opls_197	-0.0446	75
O7	opls_186	-0.08797	76

C18	opls_142	-0.16038	77
H19	opls_144	0.149807	78
C19	opls_142	-0.06037	79
H20	opls_144	0.114747	80
C20	opls_158	0.016851	81
H21	opls_140	0.138853	82
O8	opls_154	-0.5024	83
H22	opls_155	0.398825	84
C21	opls_197	-0.01832	85
O9	opls_186	-0.19984	86
C22	opls_136	-0.03608	87
H23	opls_140	0.055884	88
H24	opls_140	0.055884	89
C23	opls_136	-0.02335	90
H25	opls_140	0.051103	91
H26	opls_140	0.051103	92
C24	opls_139	-0.00214	93
C33	opls_135	-0.02875	94
H42	opls_140	0.028515	95
H43	opls_140	0.028515	96
H44	opls_140	0.028515	97
C25	opls_183	-0.02093	98
H27	opls_185	0.077924	99
C26	opls_136	-0.0092	100
H28	opls_140	0.036563	101
H29	opls_140	0.036563	102
C27	opls_136	-0.04085	103
H30	opls_140	0.031037	104
H31	opls_140	0.031037	105
C28	opls_159	0.086978	106
O11	opls_154	-0.51337	107
H36	opls_155	0.364558	108
C31	opls_136	-0.02079	109
H37	opls_140	0.035489	110
H38	opls_140	0.035489	111
C32	opls_135	-0.03589	112
H39	opls_140	0.015952	113
H40	opls_140	0.015952	114
H41	opls_140	0.015952	115
C29	opls_183	-0.02655	116
H32	opls_185	0.078993	117
C30	opls_135	-0.04185	118
H33	opls_140	0.02901	119
H34	opls_140	0.02901	120
H35	opls_140	0.02901	121
O10	opls_180	-0.24009	122
